# Fibromuscular Dysplasia Presenting as Acute Unilateral Renal Infarction: A Case Report and Review of Two Diseases

**DOI:** 10.7759/cureus.35933

**Published:** 2023-03-09

**Authors:** Amanda Abi Doumet, Brian Bustos, Jacob Garrell, Momina Salman, Lalarukh Haider

**Affiliations:** 1 Internal Medicine, University of Connecticut, Farmington, USA; 2 Primary Care Internal Medicine, University of Connecticut, Farmington, USA; 3 Nephrology, University of Connecticut, Farmington, USA

**Keywords:** systemic vascular disease, vascular disorder, management of renal artery infarction, renal artery infarction, renal fibromuscular dysplasia

## Abstract

Fibromuscular dysplasia (FMD) is a rare systemic vascular disease that has been found to present as a renal infarction (RI) in only a handful of cases.

We present a case of a 53-year-old Vietnamese patient presenting for sharp, severe left-sided abdominal pain of two-day duration associated with a migraine headache. On presentation, she was afebrile, and her vital signs were stable. Laboratory investigations were significant for mildly elevated leukocytosis but were otherwise normal. CT abdomen and pelvis with contrast revealed a left-sided renal infarct. The patient was then admitted to the hospital and started on therapeutic anticoagulation. A transthoracic echocardiogram was obtained and revealed no vegetation. CT angiography of the abdomen was pursued and was significant for mild beading within the mid-right and left renal arteries, consistent with fibromuscular dysplasia.

Our patient was diagnosed with renal infarction in the setting of fibromuscular dysplasia, a combination that has been reported only a few times. Interestingly, our patient also had mild FMD based on imaging, making it even more of an unusual cause of renal infarction. This case highlights the connection between these two diseases and the need for more studies to characterize the association between them.

## Introduction

Fibromuscular dysplasia (FMD) is a non-atherosclerotic and non-inflammatory vascular disease with a female predominance [1]. It may result in stenosis, aneurysm, dissection, occlusion, or arterial tortuosity, and clinical manifestations reflect the vascular territory affected, including hypertension, headaches, myocardial infarction, transient ischemic attack, and stroke [2]. Any arterial segment may be involved; however, the renal arteries are most commonly affected [3]. FMD has been found to present as a renal infarct in a handful of cases. We present a case of abdominal pain secondary to renal infarct resulting from FMD and review both disease entities.

## Case presentation

**Figure 1:**
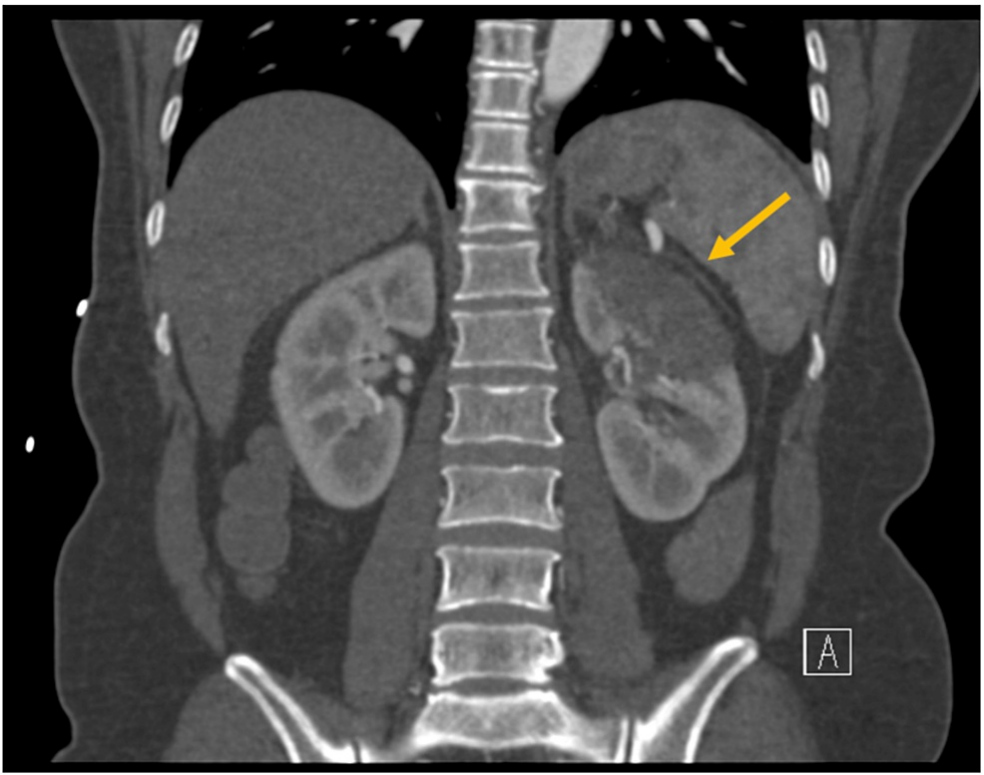
CT abdomen revealing left upper pole cortical hypoattenuation compatible with infarct with left renal segmental artery occlusion.

A 53-year-old Vietnamese female with a past medical history significant for acid reflux, iron deficiency anemia, subclinical hyperthyroidism due to Graves’ disease, and migraine headaches presented with left-sided abdominal pain. She has no history of hypertension, chronic kidney disease, or coronary artery disease. She was seen in the ED two days before admission with the same complaint. At that time, a CT scan of the abdomen revealed a non-obstructing kidney stone without any acute abnormalities. She was subsequently discharged home after the alleviation of her symptoms. One day before admission, she experienced a sudden onset migraine headache and intense pain in her abdomen. The abdominal pain was localized to the left upper quadrant, epigastric area, and left flank. It was noted to be sharp, throbbing, intermittent, and 8/10 in intensity. On presentation, she was afebrile, and her vital signs were stable. Laboratory investigations were notable for mildly elevated leukocyte count. Her creatinine was 0.8 mg/dL (normal range: 0.5-1.3 mg/dL), and her urinalysis was unremarkable. CT of the abdomen and pelvis with contrast revealed a left-sided renal infarct (Figure 1).

**Figure 2:**
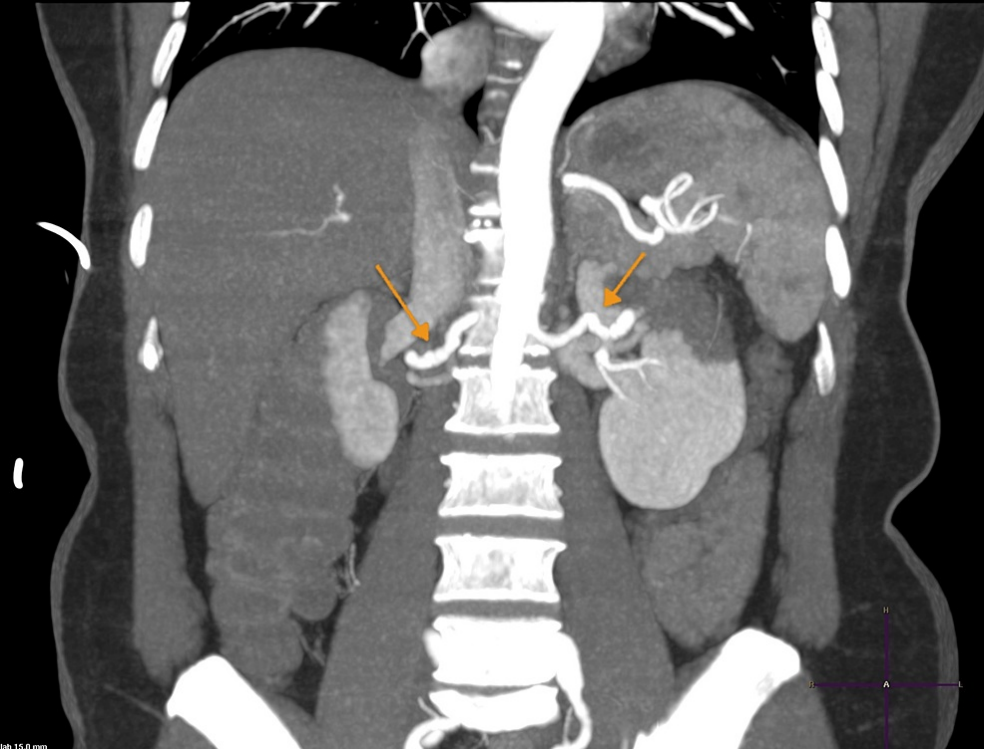
CT angiogram of the abdomen showing mild beading of the mid-right and left renal arteries and a tortuous left renal artery.

The patient was admitted for renal infarction of unknown etiology and was started on therapeutic Lovenox and ACE inhibitors for blood pressure management. She was placed on telemetry, which revealed no evidence of atrial fibrillation. Electrocardiogram showed normal sinus rhythm. A transthoracic echocardiogram revealed no vegetation, and the atria were of normal size. CT angiography of the abdomen was significant for mild beading within the mid-right and left renal arteries, consistent with FMD (Figure 2).

Lactate dehydrogenase was elevated at 1,052 U/L (normal range 105-333 U/L). A limited hypercoagulability workup was unremarkable. CT angiography of the head and neck and MRI head and neck were unremarkable. The patient remained stable during the hospital course, and her symptoms improved. She was discharged home with Apixaban 5 mg to be taken twice daily for six months, in addition to ACE inhibitors for hypertension.

## Discussion

Fibromuscular dysplasia (FMD)

FMD is an idiopathic, segmental, non-atherosclerotic, and non-inflammatory disease of the musculature of arterial walls, leading to stenosis of small and medium-sized arteries [4]. It is limited to the arterial circulation, and any arterial segment may be involved. However, the renal arteries are most affected (75%-80%), followed by the extracranial arteries (75%) [3,4]. It is more frequently diagnosed in women (82%-95%) and can occur at any age [5].

FMD was historically classified histopathologically into categories based on the layer of the artery that was involved: medial fibroplasia, perimedial fibroplasia, intimal fibroplasia, medial hyperplasia, and adventitial fibroplasia [4]. Additionally, the composition of the arterial lesion was considered, such as collagen deposition or hyperplasia of smooth muscle cells. It is no longer classified histologically due to specimens being rarely obtained [4]. In 2012, the French and Belgian Consensus proposed transitioning to an angiographic classification system since the diagnosis of FMD is now almost exclusively diagnosed through imaging [4,6,7]. The two most classified angiographic appearances are multifocal FMD and focal FMD, which have different phenotypic presentations and natural histories. Multifocal FMD describes the classic “string of beads” appearance and correlates pathologically to medial fibroplasia [8]. Whereas unifocal FMD typically correlates with intimal fibroplasia, although both medial hyperplasia and periarterial hyperplasia can also have a focal appearance. Unifocal FMD correlated with a younger age at diagnosis, earlier onset of hypertension, and increased likelihood to undergo revascularization [8,9].

The etiology of FMD still remains unknown. There have been several factors implicated, including genetic, environmental, and smoking. Familial studies have reported a minimal prevalence of 11% of documented familial renal artery FMD [10]. Genetic syndromes that involve altered TGF-Beta signaling demonstrated that there have been elevated plasma TGF-B1 and TGF-B2 and increased TGF-B1 and TGF-B2 secretion in dermal fibroblast cell lines in subjects with FMD. A study in 2016 confirmed an association between FMD and a variant in the phosphatase and actin regulator gene (PHACTR1). The intronic variant of this regulator gene, rs9349379, was also found to be associated with coronary artery disease, migraines, and cervical artery dissection [11]. Hormonal influence has also been implicated, considering the higher prevalence in adult females. Immunohistochemical staining in patients with FMD compared to FMD-free patients has detected progesterone receptor expression in nuclei of smooth muscle cells [12].

The symptoms and signs of FMD depend on the arteries involved and the severity of the arterial lesions. According to the US registry for FMD, the most frequent presenting symptoms include hypertension (63.8%), headache (52.4%), pulsatile tinnitus (27.5%), and dizziness (26.0%) [3]. Other symptoms may herald associated aneurysm or dissection, including neck pain (22.2%), tinnitus (18.8%), chest pain or shortness of breath (16.1%), or flank/abdominal pain (15.7%) [8]. The gold standard diagnostic method is catheter-based angiography. A few non-invasive imaging techniques, in contrast, have largely replaced this, including duplex ultrasound, computed tomographic angiography, and magnetic resonance angiography.

The treatment for FMD is based on the affected vessels. In this review, we will discuss the treatment of renal artery FMD. Medical therapy solely, revascularization, or surgery are the main treatment options. The initial drug of choice is an angiotensin-converting enzyme (ACE) inhibitor or angiotensin receptor blocker (ARB) to treat hypertension by targeting the underlying pathogenesis in the activation of the renin-angiotensin-aldosterone system [13]. The blood pressure goal is the same as in similarly aged hypertensive patients. Furthermore, if blood pressure is still uncontrolled, a thiazide diuretic or long-acting dihydropyridine calcium channel blocker is the next class used [6]. There have been no randomized trials comparing revascularization with medical therapy alone in patients with renal FMD; however, revascularization was shown to be beneficial in certain groups of patients with FMD, namely younger hypertensive patients with focal FMD, patients with resistant hypertension despite compliance with a three-drug regimen, patients who are unable to tolerate antihypertensive medications or are non-compliant, and patients with bilateral renal FMD or unilateral renal FMD to a single functioning kidney with progressive renal insufficiency suspected to be from renal artery stenosis [7,9].

Renal infarction (RI)

Renal infarction results from cessation of blood flow through the renal arteries, leading to ischemia and irreversible damage to the renal parenchyma. It is a rare condition with incidence rates estimated at 0.004%-0.007% [14]. Patients are usually between 60 and 70 years of age and typically present with nonspecific symptoms, including acute onset flank (50%) or abdominal pain (53%) accompanied by nausea (16.9%), vomiting (13%), and fever (10%) [14, 15]. One of the common presenting signs of RI is hypertension, which occurs due to ischemia-mediated Renin release. Other signs include lumbar tenderness and abdominal tenderness [16]. Due to its rarity and nonspecific presentation, RI is frequently misdiagnosed or diagnosed late; however, more recent studies report higher incidences, likely due to the more widespread use of abdominal CT to evaluate abdominal pain [17].

Laboratory findings include leukocytosis, elevated LDH, elevated CRP, and microscopic hematuria [14,15]. Serum creatinine may initially be normal, then increase in the few days after the event [15]. Most patients will initially undergo a non-contrast CT scan of the abdomen to evaluate for more common pathologies of abdominal pain, including nephrolithiasis, pyelonephritis, and gastrointestinal conditions. If these tests are unrevealing, the next step would be a CT angiography scan, which is the test of choice to assess for renal infarction. RI typically appears as a wedge-shaped perfusion defect and, in some cases, is accompanied by perirenal stranding due to preserved capsular circulation [15,17].

Renal infarction is most associated with cardiac conditions, especially atrial fibrillation. Other causes include valvular heart disease, ischemic heart disease, endocarditis, hypercoagulability disorders, hematologic disorders, and idiopathic [14,18]. Although atrial fibrillation was the most commonly reported risk factor, more recent studies conducted by Bourgault et al. reported the most common cause of RI to be renal artery disease (30.8%), including renal artery dissection, FMD, and Ehler-Danlos syndrome, as compared to 24.5% of RI which was attributed to cardiac origin [14]. Another study by Bolderman et al. found no evidence of cardiac disease in 59% of patients; however, did observe a high incidence of heritable thrombophilia and elevated homocysteine levels [16].

After a diagnosis of RI is made, patients should obtain an EKG, an echocardiogram, Holter monitoring, and a hypercoagulability workup to evaluate for potential etiologies [16]. The optimal treatment is debatable and is affected by many factors, including the etiology of renal infarction, ischemia time, presence of collateral blood flow, and pre-existing kidney disease [19]. Options include initiation of anticoagulation with Heparin, then transition to Coumadin or the initiation of newer-generation oral anticoagulants, surgical thrombectomy, systemic thrombolysis, and catheter-directed thrombolysis (CDT), which is more commonly reserved for patients within the first 12-24 hours after diagnosis [19]. In patients with evidence of thromboembolic disease, the most common treatment of choice is anticoagulation. Patients with an early diagnosis, evidence of bilateral ischemia, or solitary kidney should be considered for revascularization therapy, as mentioned by Sepulveda et al. [15]. 

Unfortunately, there are currently no clinical trials that have evaluated those different treatment modalities and their outcome. A study conducted by Silverberg et al. evaluating surgical revascularization or CDT revealed that even if the blood flow is restored as confirmed by CT angiography, this did not translate into improvement in kidney function [19]. In contrast, a study conducted by Sepulveda et al. revealed that four out of five patients who received curative treatment with endovascular procedures and thrombolysis, thromboaspiration, or stenting were associated with significant improvement in renal function [15]. More studies are needed to compare these different strategies and their outcomes.

The most common sequelae of RI are a decline in kidney function and persistent hypertension. Interestingly, our patient developed hypertension during her hospitalization and was initiated on ACE inhibitors for blood pressure management, in addition to Apixaban for anticoagulation.

## Conclusions

In this case report, we presented a case of renal infarction as an initial presentation of FMD, a combination that has been reported only a few times. Additionally, our patient had very mild FMD based on imaging, making it even more of an unusual cause of renal infarction. More studies are needed to outline the connection between these two conditions and the management implications.

## References

[1] Slovut DP, Olin JW (2004). Fibromuscular dysplasia. N Engl J Med.

[2] Narula N, Kadian-Dodov D, Olin JW (2018). Fibromuscular dysplasia: Contemporary concepts and future directions. Prog Cardiovasc Dis.

[3] Kim ES, Olin JW, Froehlich JB (2013). Clinical manifestations of fibromuscular dysplasia vary by patient sex: a report of the United States registry for fibromuscular dysplasia. J Am Coll Cardiol.

[4] Gornik HL, Persu A, Adlam D (2019). First International Consensus on the diagnosis and management of fibromuscular dysplasia. Vasc Med.

[5] Persu A, Dobrowolski P, Gornik HL (2022). Current progress in clinical, molecular, and genetic aspects of adult fibromuscular dysplasia. Cardiovasc Res.

[6] Olin JW, Gornik HL, Bacharach JM (2014). Fibromuscular dysplasia: state of the science and critical unanswered questions: a scientific statement from the American Heart Association. Circulation.

[7] Persu A, Touzé E, Mousseaux E, Barral X, Joffre F, Plouin PF (2012). Diagnosis and management of fibromuscular dysplasia: an expert consensus. Eur J Clin Invest.

[8] Olin JW, Froehlich J, Gu X (2012). The United States Registry for Fibromuscular Dysplasia: results in the first 447 patients. Circulation.

[9] Savard S, Steichen O, Azarine A, Azizi M, Jeunemaitre X, Plouin PF (2012). Association between 2 angiographic subtypes of renal artery fibromuscular dysplasia and clinical characteristics. Circulation.

[10] Pannier-Moreau I, Grimbert P, Fiquet-Kempf B, Vuagnat A, Jeunemaitre X, Corvol P, Plouin PF (1997). Possible familial origin of multifocal renal artery fibromuscular dysplasia. J Hypertens.

[11] Kiando SR, Tucker NR, Castro-Vega LJ (2016). PHACTR1 Is a genetic susceptibility locus for fibromuscular dysplasia supporting its complex genetic pattern of inheritance. PLoS Genet.

[12] Silhol F, Sarlon-Bartoli G, Daniel L (2015). Intranuclear expression of progesterone receptors in smooth muscle cells of renovascular fibromuscular dysplasia: a pilot study. Ann Vasc Surg.

[13] Dworkin LD, Cooper CJ (2009). Clinical practice. Renal-artery stenosis. N Engl J Med.

[14] Bourgault M, Grimbert P, Verret C (2013). Acute renal infarction: a case series. Clin J Am Soc Nephrol.

[15] Sepulveda L, Oliveira M, Oliveira A (2014). Renal infarction: three case reports and literature review. Wr Jr Neph Uro.

[16] Bolderman R, Oyen R, Verrijcken A, Knockaert D, Vanderschueren S (2006). Idiopathic renal infarction. Am J Med.

[17] Korzets Z, Plotkin E, Bernheim J, Zissin R (2002). The clinical spectrum of acute renal infarction. Isr Med Assoc J.

[18] Saarinen HJ, Palomäki A (2016). Acute renal infarction resulting from fibromuscular dysplasia: a case report. J Med Case Rep.

[19] Silverberg D, Menes T, Rimon U, Salomon O, Halak M (2016). Acute renal artery occlusion: Presentation, treatment, and outcome. J Vasc Surg.

